# Changes in mortality and human longevity in Kerala: are they leading to the advanced stage?

**DOI:** 10.3402/gha.v7.22938

**Published:** 2014-05-15

**Authors:** Muttikkal B. Thomas, Kuriath S. James

**Affiliations:** Population Research Centre (PRC), Institute for Social and Economic Change (ISEC), Bangalore, India

**Keywords:** epidemiological transition, mortality and longevity, Kerala, advance stage of mortality

## Abstract

**Background:**

During the last century, Kerala witnessed drastic mortality reduction and high improvement in longevity. This achievement is often compared with that of developed countries. However, how far the early advantages in mortality reduction have further enhanced in Kerala remains unknown. In most developed countries, advanced stage of mortality reduction and further increase in longevity was achieved mainly due to the mortality shift from adult and older ages to oldest ages (Olshansky and Ault 1986).

**Objectives:**

Considering the lack of comprehensive study on the change in longevity in Kerala, this study focuses on discovering (i) the historical time-periods that provided the biggest gain to life expectancy and also the beneficiaries (by age group and sex) and (ii) the contributions of major groups of causes of death in mortality reduction and consequent improvement in longevity.

**Methodology and data:**

The study uses the methodology proposed by Olshansky and Ault in 1986. It used methods such as Temporary Life Expectancy (TLE), Annual Relative Change in TLE, Decomposition of changes in longevity among different age groups (gender and spatial) and causes of deaths, for the analysis. It used data from various sources such as Census, Civil Registration System (CRS) and Directorate of Health Services (DHS), as well as survey data from Sample Registration System (SRS) and Medically Certified Causes of Deaths (MCCD) for this study.

**Finding and conclusion:**

The study found that overall mortality dramatically declined in the state in the recent decades. Younger ages have contributed the most for this reduction. Therefore, further mortality reduction is possible in adult and early old ages. However, the contribution of these ages to life expectancy was lower than that of youngsters until 1991–2000 especially among males. This may indicate a slow progress towards the advanced stage of epidemiological transition characterized by high prevalence of non-communicable diseases. The paper concludes that although the health issues of infants, children, and mothers in the reproductive age group, are effectively addressed through various policies in Kerala, the state needs to focus more on the health problems of adults, especially males.

In the past century, Kerala witnessed a remarkable decline in mortality and considerable advancement in life expectancy. This change was often compared to the pattern observed in developed countries (1, 2). Interestingly, Kerala has achieved low mortality despite low per capita income and higher incidence of malnourishment (3, 4). This remains a paradox to the development theorists. The changes in Kerala were highlighted as unique and referred to as the ‘Kerala Model’ of development (3, 5, 6).

There are conflicting arguments on the rapid decline in mortality and the reasons thereof. On one hand, it is postulated that state intervention since 1956 has played a major role in achieving reduction in mortality. According to a group of scholars, including Panikar and Soman, the increase in life expectancy was the result of superior medical care through primary health institutions and other measures, such as provision of clean water, sanitary facilities, and an efficient public distribution system, introduced since the formation of the state in 1956 (4, 7). The primary health institution includes primary healthcare services such as vaccination, direct medical care for infectious diseases, and perinatal, maternal, and child care, besides raising general awareness about health. In a comprehensive study, Caldwell (8) described that the role of the state was inevitable for the high rate of mortality decline in Kerala. He pointed out the role of state-supported healthcare and education system, ensuring accessibility to public health to all, universal immunization, and the provision of antenatal and postnatal services, as a noteworthy aspect in achieving a high rate of mortality decline in Kerala. Another study by Krishnan (9) also enunciated the role of the state by citing the evidence of improved health outcomes in the Malabar region after the expansion of state health facilities in that region. Contrary to this, some scholars argued that the beginning of the massive reduction in mortality in Kerala could be traced to the era before the formation of the state. Kerala was formed on November 1, 1956, by merging Travancore and Cochin, two princely states, with Malabar (a part of erstwhile Madras presidency) on a vernacular basis. They argued that social and cultural improvement, especially through education, climatic conditions, the scattered pattern of settlements, and the mysterious disappearance of a major cause of death, namely plague, were the prime factors for reduction in mortality (5, 10, 11). However, there was no detailed historical assessment of mortality reduction and increased life expectancy in Kerala that could lend clarity to this debate. Moreover, the recent changes in the pattern of mortality were not analyzed carefully.

A comprehensive assessment of mortality trends necessitates an investigation into the expected pattern of change based on the experience in developed countries. Mortality reduction is closely linked to the shifts in disease pattern. In the first stage of mortality reduction, the shift occurs in the cause of death pattern from infectious to chronic-degenerative diseases (12). This leads to a distribution of death from younger to older age groups (>50 years). However, the transition later moves from the older age groups to the oldest age group, which is also known as the age of delayed degenerative diseases (13). At this stage, there is further postponement of death in older to the oldest age groups (ages more than 80) as a result of bringing down deaths from degenerative diseases from adult ages to older ages (13). Similar stage of epidemiological transition is also later suggested by Omran where it was described as Age of declining cardiovascular mortality with ageing, life style modification as well as more death from emerging and resurgent diseases. He also predicted a fifth stage, namely Age of aspired quality of life, with paradoxical longevity and (futuristic stage) persistent inequities.

Although it is well known that Kerala has moved from younger age group mortality to older age groups, the extent of transition to the oldest age group (delayed degenerative disease pattern), and its major causes of death remain unknown. Even though various studies have shown clear dominance of lifestyle/chronic diseases as causes of death and morbidity, they were restricted only to particular social and economic groups or to a specific period of time (14–16). Nevertheless, a preliminary study was conducted by Thomas in 2012 (17) aimed at looking into the changes in mortality by using point estimates such as mortality rates and causes of death, among others, and it was found that there was a transition in mortality to the adult age groups. Similarly, causes of death shifted to chronic degenerative lifestyle diseases from infections and primary healthcare–oriented diseases. Hence, it was necessary to conduct an in-depth investigation into this problem to identify the exact contribution of different age groups to life expectancy. Secondarily, there is also necessity to identify the contribution of major causes of death to the improvement in life expectancy in Kerala.

Considering the aforementioned aspects, this paper examines the pattern of mortality changes and human longevity in Kerala since the beginning of the past century. It aims to capture the dynamics of mortality reduction over the decades to explore the levels and trends in the transition process. Further, it investigates how far Kerala has moved from older age group mortality to the oldest age group mortality as experienced in the developed countries. Specifically, the analysis attempts to establish the time-periods that provided the biggest gain to life expectancy and also the beneficiaries (by age group and sex) and the extent of mortality reduction. It was also of interest to know the contributions of major groups of causes of death in mortality reduction.

## Data and methodology

A major difficulty in measuring mortality transition in Kerala emerges from the unavailability of a single reliable data set in the past century. The only reliable data prior to the 1960s are the decennial census in India (13, 14, 18–21). However, the inception of the Sample Registration System (SRS) in Kerala provided an alternative and more reliable data on mortality after the mid-1960s. Therefore, the study mainly uses these two data sets for the purpose of estimation, that is, the first main set of data is from Monograph No: 7, Census of India 1961 (1911–20 to 1951–60) (18, 19) and rest of the data (1971–80 to 2001–08) are from the SRS. However, there was no readily available data for the period from 1961 to 70. Therefore, the study uses figures from the Western Model of Life Tables by Coale, Demeny, and Vaughan (22) for the decade 1961–70 as proxy considering the life expectancy of Kerala as estimated by Bhat (23). The study also uses data from the survey of Medically Certified Causes of Deaths (MCCD) for estimating the contribution of major group of causes of deaths in the advancement of longevity in the state. The MCCD data were obtained from the Registrar General of India's Report on Medical Certification of Cause of Death for various years, published by the Office of the Registrar General and Census in New Delhi.

It is understandable that the data used for the study are limited by their quality. The census data used to analyze the patterns of mortality are constrained by the inaccuracy in the mortality estimations due to two factors. First, to make reliable estimates of mortality from census data, a supplementary data on infant and child mortality are essential. These are not always readily available. Second, the census data are often subject to bias due to rampant misreporting of age by the respondents (13). Similarly, the proxy mortality rates taken from the ‘Western Model of Life Table’ may have slight variations between different age intervals. At the same time, the quality of mortality data from SRS may also be restricted by their own sample size. Likewise, the data from MCCD are also handicapped by their lack of quality and non-availability. Considering this fact, this study uses information on causes of death from MCCD that is available from 1976 for urban areas in the state.

The paper follows the methodology forwarded by Olshansky and Ault (16) considering the possibility of mortality reduction from adult and early old age groups to the oldest age group. It compares the change in absolute value of mortality level by the increase or decrease in life expectancy between different periods. However, a major lacunae of life expectancy indicator is that it measures the mortality level for an open age interval x and above. Therefore, it is often limited to the problems of data reliability in older age groups and the restriction on limits of human life span (24). To avoid these problems, the paper analyses the relative risk of mortality transition for closed age interval (x, x+n) by using Temporary Life Expectancy (TLE) and index of Annual Relative Change (ARC) of TLE in the second section. Finally, the paper analyses changes in relative importance of death in older age groups in Kerala by estimating the rate of survival to older age groups, median age of death, and the exact contribution of each group (by age and sex) toward life expectancy at different time intervals. The study also decomposed the improvement in life expectancy by major group of causes of death to identify the dominance of major diseases.

For the purpose of analysis, the study classifies the male and female population into three major groups as youngsters (aged 0–15 years), adults (aged 15–60 years), and old (>60 years). However, the adults and the aged population are again sub-divided into young adults (aged 15–40 years), old adults (aged 40–60 years), older age group (aged 60–80, and the oldest age group (aged 80+) for cross examination while considering the vulnerability of diseases (25, 26). Similarly, the historical periods are also divided into different decadal intervals.

## An overview of changes in death rates and life expectancy in Kerala

### Magnitude of absolute change in life expectancy

Through the reduction in mortality rates, an impressive level of life expectancy has been achieved in Kerala since 1911–20. Such a change can be seen over the time periods as well as among the age and sex groups. Table 1 records the levels and changes in the life expectancy in the state over the past century. It shows that the life expectancy at birth of males rose from 25.5 years in 1911–20 to 70.7 years in 2001–05. Similarly, female life expectancy increased from 27.4 years to 77.1 years. This improvement gave an advantage of 45.2 years for males and 49.7 years for females within 88 years of time – an average annual increase of about 0.51 and 0.56 years, respectively. However, the average annual increase within each decade reflects disparities among the decades, that is, the pace of increase in life expectancy was not constant but varied from decade to decade as shown in the Table 1. Except in 1951–80 for both sex groups and 1921–40 for males and 1971–90 for females, the annual contribution of absolute years to life expectancy was below the overall average showing a low pace in those decades. However, the two decades of 1951–60 and 1961–70 recorded a high pace in absolute changes of life expectancy. Perhaps, the actual value of the increment will be more in terms of relative changes considering restriction due to the limits of human life span (24).

**Table 1:** Levels and changes of life expectancies at birth in Kerala during 1911–20 to 2001–08

Period		Absolute increase		Annual average years added	
		
From	To	Male	Female	Male	Female
1911–20	2001–08	45.25	49.71	0.51	0.56
1911–20	1921–30	4.05	5.29	0.41	0.53
1921–30	1931–40	5.49	5.22	0.55	0.52
1931–40	1941–50	4.58	5.06	0.46	0.51
1941–50	1951–60	4.63	5.17	0.46	0.52
1951–60	1961–70	9.90	9.41	0.99	0.94
1961–70	1971–80	7.83	7.10	0.78	0.71
1971–80	1981–90	4.53	7.64	0.45	0.76
1981–90	1991–00	2.68	2.76	0.27	0.28
1991–00	2001–08	1.56	2.07	0.19	0.26

Source: Calculated from Namboodiri, 1968; Coale, Demeny and Vaughan, 1983; Bhat, 1987; SRS various years.

Similarly, the magnitude of changes in life expectancy is different among the age groups, as shown in Table 2. The early decades registered a high, absolute change in life expectancy in the younger age groups. Nevertheless, a slowdown in gains in life expectancy in the younger age groups and comparatively high gains in life expectancy in the advanced age groups are visible in the recent. For instance, the absolute change in life expectancy for females at birth during 1911–20 to 1941–50 was 15.6 years (56.9%) while it was 4.1 years (48.8%) at age at 60. But in recent decades (1971–80 to 2001–08), the absolute changes in life expectancy at birth was by 12.4 years (19.2%), 4.9 years (30.6%) at age 60, and 2.6 years (49.1%) at age 80 in Kerala. This indicates a major shift in the relative gain at different ages toward increase in life expectancy. There is an obvious reversal of gains in life expectancy at the younger ages has given way to a drastic increase in the life expectancy at the advanced ages. Nevertheless, this change in advanced age groups was relatively lower among males than females. Between 1971–80 and 2001–08, the life expectancy of males increased only by 8.7 years at birth (14.0%), by 2.4 years (16.0%) at age 60, and by 1.6 years (30.2%) at age 80 indicating delay in mortality transition among them.

**Table 2:** Life expectancy at different age levels in Kerala during 1931–40 to 2001–08, by sex

	1911–20	1921–30	1931–40	1941–50	1951–60	1961–70	1971–80	1981–90	1991–00	2001–08
Male										
0	25.5	29.5	35.0	39.6	44.2	54.1	62.0	66.5	69.2	70.7
15	29.6	33.8	35.5	38.5	41.5	49.0	53.2	55.0	55.9	57.0
40	15.1	17.0	18.4	20.6	22.7	28.6	30.4	32.0	32.7	33.7
60	6.9	8.6	9.7	10.6	11.5	14.4	15.0	16.6	16.7	17.4
80	–	–	3.8	4.0	4.3	5.1	5.3	6.6	6.9	6.9
Female										
0	27.4	32.7	37.9	43.0	48.1	57.6	64.7	72.3	75.1	77.1
15	28.9	33.7	36.8	40.3	43.6	51.6	56.3	60.6	61.6	63.4
40	16.6	17.8	22.4	24.3	26.3	31.2	33.3	37.0	37.8	39.4
60	8.4	9.0	11.5	12.5	13.4	16.0	16.3	19.2	19.9	21.2
80	–	–	4.2	4.5	4.8	5.6	5.3	6.9	7.3	7.9

Source: Calculated from Namboodiri (18); Coale, Demeny, and Vaughan (22); Bhat (23); SRS various years.

The sex difference in life expectancy, especially at birth, is also a notable feature of life expectancy changes in Kerala as indicated in Table 2. The table shows a narrow level of discrepancy in life expectancy at birth during the early decades, which widened in the more recent decades. For instance, in 1911–20, the sex difference at birth was only 1.9 years and rose to 6.4 years in the 2001–08 period. It is important to note that this sex difference in life expectancy has remained at a higher level since 1981–90, with females surging ahead in gaining greater mortality decline and improvement in life expectancy than males. This change is very different from the US experience where the sex difference in life expectancy was projected to end in the fourth stage of epidemiological transition (13). In short, the analysis of absolute change in life expectancy shows that life expectancy in Kerala has improved over the decades in absolute terms. However, there are differences in the magnitude of such changes across the time periods as well as among age and sex groups.

The absolute life expectancy estimates, which can be used as a proxy to the rate of mortality, suffer from problems of relative magnitudes. In other words, the possible change in life expectancy indicators depends on the level of already achieved life expectancy (24). Moreover, the estimation of life expectancy is constrained by technical problems of unreliability of data, especially pertaining to the older age groups (generally more than 65 years) in the developing countries (25). Besides, there is a possibility of a counteracting effect, which neutralizes the contribution of different age groups – a high mortality increase experienced by one group is offset by a reduction in the other group (27). This point to a certain level of arbitrariness in results (with respect to mortality changes) which was estimated from the absolute life-expectancy indicators. These lacunae in estimation call for an alternative measure to capture the changes in mortality considering its relative risks at different age structures, life expectancy levels, and the quality of data at different age groups. Therefore, we use TLEs and the index of ARC of TLE for further analysis.

## Transition in relative risk of mortality by age and sex

### Temporary life expectancies

Table 3 shows TLE at select exact age intervals by sex for Kerala between 1931–40 and 2001–08. The TLE (*i*^*e*^*x*) from age x to (x+i) is the average number of years that a ‘group of persons’ alive at exact age x will live from age x to (x+ i) years (24)

i.e.exi=(Tx-Tx+ilx)

where, *i*^*e*^*x* represents the TLE from age *x* and *x*+ n. *T*_*x*_ and *T*_*x*+*i*_ are the number of person-years lived at age *l*_*x*_ and older, and *x*+*i* and older, respectively. *l*_*x*_ is the number of survivors at age *x* in a life table (as radix) of 100,000.

**Table 3:** Observed temporary life expectancies at selected exact age intervals by sex (1931–40 to 2001–08)

	1931–40	1941–50	1951–60	1961–70	*1971–80	*1981–90	*1991–00	*2001–08
Male								
0–80	34.96	39.49	44.01	53.40	60.95	64.76	67.24	68.56
0–15	10.85	11.48	12.06	12.94	13.79	14.34	14.69	14.77
15–40	22.12	22.61	23.04	23.71	24.46	24.53	24.60	24.62
40–60	14.32	15.29	16.11	17.88	18.45	18.54	18.77	18.89
60–80	9.33	10.11	10.88	13.03	13.48	14.23	14.20	14.70
Female								
0–80	37.75	42.69	47.68	56.41	63.30	69.49	71.82	73.13
0–15	11.43	12.04	12.62	13.20	13.77	14.42	14.73	14.78
15–40	21.58	22.21	22.76	23.81	24.52	24.68	24.74	24.78
40–60	15.88	16.47	17.11	18.34	19.06	19.38	19.44	19.57
60–80	10.87	11.66	12.39	14.13	14.49	15.94	16.22	16.74

Source: Calculated from Namboodiri (18); Coale, Demeny, and Vaughan (22); Bhat (23); SRS various years.

* The findings from 1971 onward have been taken from ref. 17.

Table 3 records a substantial increase in TLE over the decades in Kerala. However, the nature and pace of this change are different across age intervals as well as decade intervals. It can be observed that the TLE between birth and age 80 increased to 68.56 years and 73.13 years in the 2001–08 period for both males and females, respectively. It means that male babies born in Kerala during the 2001–08 period can be expected to live 68.56 years, whereas female babies can be expected to live 73.13 years, provided the mortality rates in the period when they were born remains constant throughout their lives. Nevertheless, though the expected lifespan in Kerala has almost doubled during this period, it still leaves room of 11.44 years and 6.87 years, for both male and female groups, respectively, for further improvement.

The change in TLE was also different among various age groups. It shows that younger age groups benefited more from the TLE changes than the older age groups over the decades. It should be noted that the subgroups (0–15, 15–40, 40–60) had already attained almost maximum TLE (difference was below 0.5 years) before 1971–80, except for males in the age group of 40–60. Also, though the older age group (60–80) achieved improvement in TLE, there is still ample room for improvement. It also indicates that the possibility of further mortality reduction is concentrated in the older age groups (40–60, 60–80). Moreover, it should be noted that the changes in TLE showed a severe stagnation for males, especially in these age groups in the past four decades in Kerala.

Though the data show an improvement in TLE in almost all age groups over the decades, the changes are similar for both sexes across the different age groups. Difference between the TLE at birth and age 80 can be taken as an instance where it shows a difference of 4.57 years in 2001–08. It also shows that there is a miniscule sex difference in the younger age groups (0–15, 15–40) and high disparities in the older age groups (40–60 and 60–80). It denotes that the overall difference in TLE between the ages 0 and 80 years would be due to the disparities between males and females in the older age groups. It is also worth mentioning that the reduction in difference in TLE in the younger age groups could be a result of effective control over the causes of death in the younger age groups. Perhaps, this has not happened in the case of the older age groups. A comparatively lower TLE for males than females (females almost near the maximum) in the age group 40–60 years and 60–80 years means that the mortality shift was unequal and that more males than females lagged in those age groups.

## Index of ARC of in TLE

The index of ARC in TLE during 1931–40 to 2001–08 in Kerala is recorded in Table 4. It represents the percentage change in two mortality measures in their observed reduction in deaths in relation to the total possible reduction (24). In other words, it shows the change in number of years lived between two periods considering the maximum possibility of reduction in that age group.

**Table 4:** Annual Relative Change Index of TLE at selected age intervals by sex in between different decades (1931–40 to 2001–08)

	1931–40∣ 1941–50	1941–50∣ 1951–60	1951–60∣ 1961–70	1961–70∣ 1971–80	*1971–80∣ 1981–90	*1981–90∣ 1991–00	*1991–00∣ 2001–08
Male							
0–80	1.05	1.18	2.98	3.28	2.21	1.76	1.36
0–15	1.62	1.78	3.52	5.14	5.89	7.30	3.68
15–40	1.86	1.96	4.10	8.38	1.33	1.60	0.73
40–60	1.86	1.90	5.88	3.09	0.57	1.71	1.31
60–80	0.76	0.81	2.65	0.66	1.22	−0.06	1.12
Female							
0–80	1.24	1.43	3.10	3.40	4.52	2.47	2.16
0–15	1.86	2.17	2.73	3.71	7.25	7.52	2.42
15–40	1.99	2.18	6.14	8.77	3.94	2.01	2.24
40–60	1.52	1.99	5.40	5.53	4.12	0.88	3.46
60–80	0.89	0.92	2.56	0.64	3.01	0.70	1.83

Source: Calculated from Namboodiri (18); Coale, Demeny, and Vaughan (22); Bhat (23); SRS various years.

* The findings from 1971 onward have been taken from ref. 17.

The ARC can be calculated as: AiRCxn=[1-(1-RiCxn)1n]⋅100 , where AiRCxn represents Index of ARC in TLE; *n* is the number of years, and RiCxn is the observed change in TLE in relation to the maximum possible change,

where RiCxn can be calculated as, RiCxn=ext+ni-extii-exti , where ext+ni-exti is the absolute change of TLE of years of life between two particular ages; *i* is the maximum possible TLE between the age intervals.

The figures in Table 4 indicate high variations in the pace of improvements in TLE across sex and time-period. By and large, it can be said that significant changes happened between 1951–60 and 1981–90. It may be recalled that our analyses of Crude Death Rates (CDR) and absolute changes in life expectancy have pointed to a similar effect for the period after state formation. Almost all the ages and sex subgroups were at their best performance in terms of TLE in that period. It could be due to the provision of better healthcare measures that responded to the causes of death at different time-periods in Kerala.

However, the performance of ARCs in TLE indices is negligible in the recent decades. This rapid decline in pace, especially in younger age groups, may be due to the fact that the TLE in younger age groups have rapidly approached the size of age interval and the limits of further decline. However, it is also seen in Table 7 that the ARC in TLE in the advanced age groups for both sexes (40–60 and 60–80) was comparatively lower than that of the early age groups after 1971–80 in most cases. This is interesting because we expected more changes in the older age groups considering the possibility of the advanced mortality reduction indicating a shift of mortality from the older to the oldest age groups.

Sex difference in pace of improvement in TLE is another issue of concern. It was recorded that in all the periods except 1951–60 to 1971–80 at the 0–15 and 60–80 age groups, 1981–90 to 1991–2000 at the 40–60 age group, and 1991–2000 to 2001–08 at the 0–15 age group, the pace of change was higher for females than males. Moreover, though the difference in the pace was low in 1931–40 to 1941–50, it stabilized thereafter at a higher level till 1981–90. Later, it dropped further for almost all age groups, then picked-up again in the 2001–08 period. This phenomenon was more intensive in the younger age groups than the in the older age groups. Nevertheless, the low performance of pace indices of TLE among older age groups, especially with regard to males than females, may be an indication of a lag in moving toward the stage of advanced mortality reduction in Kerala.

To sum up, the analysis of changes in the relative risk in mortality reflects an impressive change in TLE in Kerala during the past century. It was noted that the younger age groups contributed almost their maximum capacity to TLE by the reduction in mortality. However, the contribution was low from the older age groups which differed from our expectation. Moreover, there were high disparities prevalent between males and females in TLE, especially in the older age groups. This could be due to a low rate of increase in male TLE during those periods. Similar finding is also evident in the annual index of TLE. Notably, there was low rate of change in the indices of TLE after 1981–90 in most cases in advanced age groups.

## Changes in relative importance of mortality at older ages

The epidemiological transition theory put forward by Olshansky and Ault (13) emphasized the importance of mortality changes in the older age groups. In order to analyze the importance of mortality in the older age groups, this section attempts to discuss the changes in the median age of death and the proportion of survival to older age groups. Further, we analyze the contribution of each age group toward increments in life expectancy to identify the recent dynamics of mortality change and thereby understand the possibility of advanced stages of epidemiological transition.

## Median age at death and proportion survival to older age groups

Over the decades, it has been observed that the proportion of survival of population from birth to the oldest age group has increased in Kerala. Table 5 records sex-wise distribution of the proportion of survival to ages 60 and 80, and median age at death in the state during the past century. It can be observed in Table 5 that the median age of death is being pushed toward the older age groups in Kerala. Notably, the median age of death was 20.82 for males and 26.03 for females in the early decades of the century but rose to 73.85 and 80.03 for males and females, respectively, in the recent decades.

**Table 5:** Median age at death and proportion of survival to age at 60 and 80, by sex in Kerala during 1911–20 to 2001–08

	1911–20	1921–30	1931–40	1941–50	1951–60	1961–70	1971–80	1981–90	1991–00	2001–08
Median age at death										
Male	20.82	30.67	39.55	45.46	50.92	63.23	68.96	71.29	72.30	73.85
Female	26.03	35.14	39.19	47.47	55.31	67.32	72.40	77.21	78.38	80.03
Proportion surviving to age 60										
Male	9.67	15.08	20.86	28.27	36.13	55.46	68.46	73.27	77.66	80.39
Female	11.75	17.77	27.60	35.73	44.16	61.59	75.53	84.85	88.27	90.42
Proportion surviving to age 80										
Male	0.22	0.97	1.80	3.21	5.25	14.44	19.26	26.27	28.02	31.56
Female	0.73	1.36	3.95	6.41	9.65	20.44	25.49	40.75	44.61	50.26

Source: Calculated from Namboodiri (18); Coale, Demeny, and Vaughan (22); Bhat (23); SRS various years.

Similarly, the proportion of survival to the age of 60 and 80 from birth has also increased considerably over the decades in the past century. Table 5 shows that the proportion of survivors into the age of 60 was 9.67 for males and 11.75 for females in 1911–20 and increased to 80.39 and 90.42, respectively, in 2001–08 – approximately an eight-fold increase for both sexes. A similar increase is also seen in the proportion of people who survived to the age of 80 (the oldest age group). It may be noted that the survival to the oldest age group from birth cohorts in 1911–20 was less than 1%, but it increased to more than 30 percent in 2001–08 for both the sexes. The proportion of population who live for more than 60 years has dramatically increased over the century, thereby pushing mortality to the older age groups in the recent decades.

However, the pace of change in median age of death and the survival ratios vary across decades as well as by sex group. It can be observed that the rate of increase in both the median age of death and the survival ratio were relatively lower in the recent decades than in the early decades for both sexes. It could be due to low contribution from older age groups, whereas the expectations from the younger and adult ages are low because they are already close to maximum reduction. However, males have recorded relatively lower median age of death, lower survival to old ages, and comparatively lower rate of growth compared to females in Kerala. In a nutshell, the changes in proportion of survival to the older ages and the median age of deaths in the state indicates an increasing relevance of elders and their mortality to the ongoing improvement in life expectancy in Kerala. However, these indicators are incapable of revealing the exact contribution of the different age groups to the decline in mortality, which in turn bring about changes to life expectancy, limiting the possibility of understanding the advanced stage of mortality decline.

## Contribution from each age group to gains in longevity

Decomposition of contribution of mortality decline from different age groups to the gain in life expectancy is one of the best measurements for identifying the advanced stage of mortality decline and human longevity in Kerala. According to the theory of epidemiological transition, at the fourth stage, we expect a higher contribution of life expectancy from the older age groups when the life expectancy increases to its maximum. Therefore, it is important to assess the relative contribution of age groups at different periods in order to ascertain the pattern of mortality change. The percentage contribution of each age group in gain in life expectancy at different periods is shown in Table 6.

**Table 6:** Contribution of mortality change at selected ages to total change in life expectancy by sex in Kerala in between different decades, 1931–40 to 2001–08 (%)

	1931–40∣ 1941–50	1941–50∣ 1951–60	1951–60∣ 1961–70	1961–70∣ 1971–80	1971–80∣ 1981–90	1981–90∣ 1991–00	1991–00∣ 2001–08
Male							
LE +	4.58	4.63	9.90	7.83	4.53	2.68	1.56
0–15	54.7	53.0	41.5	54.6	63.8	69.6	28.3
15–40	21.5	21.1	20.9	28.0	6.4	9.1	7.0
40–60	19.6	20.2	27.2	13.1	5.1	18.9	29.6
60–80	4.1	5.6	10.0	3.9	19.1	−0.5	35.9
80+	0.1	0.2	0.4	0.3	5.6	3.0	−0.9
All	100	100	100	100	100	100	100
Female							
LE+	5.06	5.17	9.41	7.10	7.64	2.76	2.07
0–15	50.6	50.1	30.6	43.3	49.5	64.1	16.2
15–40	31.0	27.2	35.9	33.7	9.0	8.4	9.0
40–60	13.0	15.9	21.6	20.5	12.2	7.9	20.6
60–80	5.2	6.4	11.1	3.4	24.0	14.1	39.3
80+	0.2	0.4	0.8	−0.8	5.3	5.4	14.8
All	100	100	100	100	100	100	100

Source: Calculated from Namboodiri (18); Coale, Demeny, and Vaughan (22); Bhat (23); SRS various years using the formulae given by Preston et al. (28).

Kerala experienced high improvement in life expectancy in the decades of 1951–60 to 1971–80 as shown in the table. The positive values indicate the percentage gains in life expectancy due to decline in mortality. Similarly, the negative values indicate reduction in life expectancy due to increase in mortality. However, we have ignored the negative figures from the mainstream interpretation because they are very few, of small magnitude, and refer to the oldest age group where misreporting of age at death is relatively high. The improvement peaked during 1951–70 gaining more than 9.5 years of life expectancy. During this period, all age groups contributed significantly. However, the contribution of the younger age groups (0–15) was the highest. It is visible that in all periods, except for the recent one, the younger age group (0–15) is the most important contributor to life expectancy improvement. This contribution of this group ranges from 28.3 to 69.6% for males and 16.2 to 64.1% for females. Nevertheless, their contribution was comparatively higher in the 1981–90 to 1991–2000 period but declined in the 1991–2000 to 2001–08 period.

However, the contribution of the age group 15–40 varies from period to period. It is seen that the contribution was high (between 20.9 and 35.9%) until 1971–80 but significantly came down (between 6.0 and 9.1%) after this period for both the sexes. The drastic change could be due to the fact that this age group had attained almost the maximum possible reduction during those decades. However, the age group of 40–60 years shows an increasing pattern in the contribution to life expectancy over the decades for both the sexes. Their relative contribution was below 20 percent in 1931–40 to 1941–50 but was significantly higher during the 1991–2000 to 2001–08 period. Interestingly, the contribution of the oldest age group (60–80) shot up from a nominal share (below 5%) to >35% during the same period.

It must be noted that the data for 2001–08 present a different picture. Though the values are small and the period does not represent a decade, the figures show a reversal from the previous trend. It indicates a high contribution of improvement in life expectancy from the oldest age group than from the youngsters as a result of greater reduction in mortality among adults and the older age groups when compared with the youngsters. Therefore, the emerging trends may indicate the beginning of a fourth stage of mortality reduction in Kerala as it has happened in developed countries. Moreover, the new trend is more evident in females than in males; the females are in advanced momentum of changes in life expectancy, whereas males are slow in decline in mortality during their adulthood and in the older age groups.

## Contribution to total changes in life expectancy by causes of death

The total changes in the life expectancy, absolute as well as its percentage contributions of years by causes of deaths to total changes in the life expectancies at selected exact ages in urban areas in Kerala are given in Tables 7 and 8. Table 7 shows that life expectancy in Kerala has been increased by 7.92 years for males and 11.13 years for females during the period 1976 to 2000–04 in urban areas. Notably, all the groups of causes of death have contributed positively to these changes. However, higher share of the contribution is from infectious and parasitic diseases which was contributed 32 and 24 for percentage respectively for both males and females during this period. Also, contribution by non-communicable diseases, accidents, and injuries is commendably low in the same period.

**Table 7:** Contribution of mortality changes by causes of deaths to the total increment in life expectancy in 1976 to 2000–04, Kerala* (Urban)

	Gain in LE	Infectious and parasites	Circulatory diseases	Neoplasm	Respiratory diseases	Digestive diseases	Endocrine, nutritional, metabolic diseases	Accidents, poisonings and injuries	Others∧
Males (1976 to 2000–04)									
0–1	1.66	0.52	0.00	0.01	0.26	0.08	0.10	0.01	0.43
%	100.00	31.52	0.14	0.32	15.68	4.85	5.92	0.31	25.68
1–4	1.08	0.34	0.01	0.01	0.15	0.07	0.05	0.01	0.22
%	100.00	31.17	0.82	1.05	14.19	6.10	4.78	0.81	20.62
5–14	0.39	0.18	−0.01	0.02	−0.01	0.06	0.01	0.00	0.06
%	100.00	46.38	−3.82	5.89	−1.60	14.86	3.02	−0.12	14.40
15–44	1.19	0.37	−0.01	0.11	−0.03	0.16	0.05	0.03	0.16
%	100.00	30.90	−0.64	9.60	−2.34	13.03	4.21	2.84	13.41
45–64	2.28	0.67	0.48	0.15	−0.01	0.19	0.06	0.02	0.16
%	100.00	29.55	21.05	6.65	−0.64	8.33	2.84	0.95	6.82
65+	1.30	0.46	0.01	0.18	−0.21	−0.03	−0.15	0.12	0.04
%	100.00	35.26	0.92	13.70	−16.39	−2.16	−11.27	8.93	2.79
All	7.91	2.54	0.48	0.48	0.15	0.52	0.13	0.19	1.06
%	100.00	32.16	6.08	6.12	1.91	6.59	1.64	2.34	13.37
Females (1976 to 2000–04)									
0–1	2.26	0.70	0.03	0.01	0.35	0.11	0.09	0.01	0.60
%	100.00	30.92	1.40	0.27	15.57	4.74	3.87	0.64	26.67
1–4	1.92	0.69	0.03	0.02	0.25	0.10	0.07	0.02	0.40
%	100.00	35.68	1.82	0.92	13.17	5.00	3.56	1.04	20.92
5–14	0.53	0.16	0.01	0.02	0.03	0.05	0.01	0.03	0.11
%	100.00	29.67	2.63	3.66	5.40	9.87	2.30	5.14	21.00
15–44	1.21	0.18	0.11	0.07	0.01	0.19	0.01	−0.06	0.21
%	100.00	14.57	8.83	5.94	0.73	15.40	0.71	−5.05	17.42
45–64	2.28	0.40	0.66	0.35	0.05	0.12	0.08	0.03	0.21
%	100.00	17.55	29.13	15.53	2.22	5.17	3.51	1.33	9.23
65+	2.92	0.57	0.35	0.17	−0.18	−0.15	−0.18	0.36	0.64
%	100.00	19.60	12.09	5.81	−6.08	−5.07	−6.18	12.43	21.99
All	11.13	2.69	1.21	0.64	0.52	0.41	0.08	0.39	2.18
%	100.00	24.19	10.83	5.74	4.63	3.70	0.68	3.55	19.60

Source: Calculated from the compiled figures of medically certified causes of deaths in 1976 and 2000–04 of Trivandrum, Kochi, Kollam, and Kozhikode Corporations and Alappuzha municipality.

* Figures are unadjusted for non-classification of causes of deaths

∧ comprise other diagnosed causes of death.

The mortality change of a specific cause may differ from one period to another and therefore, the contribution of this cause may also vary with time as shown in Table 8. The table shows the changes in life expectancy by the contribution of different groups of diseases in the urban areas of Kerala for two time-periods (from 1976 to 1990–94 and from 2000 to 2004). It is reflected that the life expectancy in Kerala has improved by 5.95 years for males and by 8.59 years for females in 1976 to 1990–94 periods in Kerala while it is only 1.97 years and 2.54 years, respectively, for males and females between 1990–94 and 2000–04. Between 1972 and 1990–94, the contribution by the infectious and parasites groups was comparatively higher for both males (1.86 years) and females (2.06) in the urban areas. However, between 1990–94 and 2000–04, the contribution from this group dwindled for both males (0.74 years) and females (0.84 years).

**Table 8:** Absolute contributions of mortality changes by causes of deaths to the total increment in life expectancy in 1976 to 1990–94 and 1990–94 to 2000–04, Kerala* (Urban)

Cause of death	Gain in LE	Infectious and parasites	Circulatory diseases	Neoplasm	Respiratory diseases	Digestive diseases	Endocrine, nutritional, metabolic diseases	Accidents, poisonings and injuries	Others∧
Male (1976 to 92)									
0–1	1.33	0.44	0.02	0.01	0.23	0.07	0.09	0.01	0.21
1–4	0.90	0.24	0.02	0.01	0.14	0.05	0.05	0.00	0.18
5–14	0.50	0.17	0.04	0.03	0.01	0.05	0.01	0.02	0.08
15–44	0.51	0.22	−0.05	0.08	−0.06	0.03	0.03	−0.26	0.14
45–64	1.28	0.46	−0.04	0.12	−0.04	−0.03	0.07	−0.12	0.31
65+	1.42	0.33	−0.60	0.46	−0.01	−0.14	0.00	−0.02	0.48
Total	5.95	1.86	−0.61	0.70	0.27	0.04	0.25	−0.37	1.41
Male (1992 to 2000–04)									
0–1	0.30	0.08	−0.01	−0.01	0.02	0.01	0.00	0.00	0.22
1–4	0.16	0.10	−0.02	0.01	0.01	0.01	0.00	0.01	0.04
5–14	−0.13	0.01	−0.05	−0.01	−0.02	0.00	0.00	−0.02	−0.03
15–44	0.69	0.16	0.04	0.04	0.03	0.13	0.02	0.27	0.03
45–64	1.07	0.22	0.57	0.04	0.03	0.24	−0.01	0.16	−0.18
65+	−0.13	0.17	0.80	−0.36	−0.28	0.15	−0.20	0.18	−0.59
Total	1.97	0.74	1.32	−0.30	−0.20	0.53	−0.18	0.58	−0.51
Female (1976–92)									
0–1	1.85	0.61	0.04	0.01	0.31	0.09	0.08	0.01	0.35
1–4	1.49	0.51	0.03	0.02	0.17	0.07	0.06	0.00	0.31
5–14	0.47	0.12	0.04	0.02	0.03	0.04	0.01	0.02	0.10
15–44	1.00	0.25	0.09	0.04	0.07	0.08	0.02	0.04	0.21
45–64	1.06	0.16	0.11	0.28	0.03	−0.01	−0.03	−0.04	0.19
65+	2.71	0.41	−0.73	0.38	0.17	−0.13	0.02	0.19	1.09
Total	8.59	2.06	−0.42	0.74	0.78	0.14	0.15	0.20	2.25
Female (1992 to 2000–04)									
0–1	0.35	0.07	−0.01	0.00	0.03	0.01	0.01	0.00	0.25
1–4	0.41	0.17	0.01	0.00	0.08	0.02	0.01	0.02	0.09
5–14	0.06	−4.91	7.07	0.25	1.49	−1.89	−0.63	−1.32	−0.16
15–44	0.18	0.08	−0.09	−0.01	0.00	0.04	0.01	0.01	0.14
45–64	1.30	0.26	0.62	0.07	0.02	0.14	0.13	0.08	0.01
65+	0.24	0.21	1.45	−0.29	−0.46	−0.02	−0.27	0.23	−0.61
Total	2.54	0.84	1.93	−0.23	−0.34	0.22	−0.12	0.37	−0.12

Source: Calculated from the compiled figures of medically certified causes of deaths in 1976 and 2000–04 of Trivandrum, Kochi, Kollam, and Kozhikode Corporations and Alappuzha municipality.

* Figures are unadjusted for non-classification of causes of deaths

∧ comprise other diagnosed causes of death.

The contribution from circulatory and digestive diseases, and accidents and injuries increased in the latest period. The circulatory diseases, which were making a negative contribution to the life expectancy in 1976 to 1990–94, became a positive contributor in 1990–94 to 2000–04, with a contribution of 1.32 years for males and 1.94 for females. On the contrary, in 1990–2004, the contributions of neoplasm (only females), endocrine, and nutritional and metabolic diseases were negative, whereas they were contributed positively between 1976 and 1990–94. Though the contribution of males and females are roughly similar, females have higher contribution in most of the groups, especially from circulatory diseases. Also, the low contribution of females toward neoplasm, and respiratory and digestive diseases are a matter of concern.

The table 8 also indicates contributions made by each cause to changes in life expectancies at different age groups that greatly vary among both males and females. The contributions by infectious diseases and parasites to life expectancy improvement were slightly higher among the younger age groups between 1976 and 1990–94. But this difference disappeared in the recent period (1990–94 to 2000–04) due to miniscule contributions from all age groups. Also, contributions of major causes of deaths such as circulatory diseases increased considerably in the recent period. The overall contribution of circulatory diseases was negative in the 1976 to 1990–94 period, mainly due to low or even negative contributions from the adult and the older age groups. However, in contrast, the recent period witnessed a positive turn in their contribution to life expectancy, which is considerably increasing with ages. Almost similar change is also visible in the accidents, poisoning, and the injuries group. In contrast, the contribution of neoplasm and respiratory diseases came down to a negative level in the older age groups between 1992 and 2000–04.

In short, it is understandable that the improvement in life expectancy slowed down in the recent decade in Kerala. Though the contribution of major cause of deaths like infectious and parasites, circulatory diseases contributed positively, it was not significant enough to move up the entire life expectancy to the highest ages of life span. Also, contributions of other major groups such as neoplasm and respiratory diseases worsened. However, a tendency of increasing contributions to the life expectancy from the oldest age groups is visible reflecting a movement toward the advanced stages mainly among females.

## Discussion

A major focus of the paper is to identify the period during which mortality transition and improvement in human longevity significantly happened in Kerala. Our analysis found that, though Kerala has experienced a drastic decline in mortality and a resultant impressive growth in life expectancy throughout the past century, the major reduction occurred between 1951 and 1970. Decline in child and infant mortality rates has played an important role during the period, resulting in drastic reduction in mortality. These findings corroborate with that of others, especially Caldwell, who pointed out the historical time period when the state had experienced the highest mortality reduction by using macro level indicators. Moreover, such corroboration also nullifies the arguable inconsistency in the core trend in mortality decline that stems from usage of different data sets for estimation over a centurial period. Also, the higher reduction of mortality achieved through a reduction in infant and child mortality was the result of healthcare intervention by the state through effective primary healthcare programs such as building awareness, vaccination, and so on, as pointed by the scholars. This draws parallels between Kerala's experience and that of other developing countries where primary healthcare has been more dominant than the socioeconomic improvements in the early stages of mortality reduction. Moreover, it upholds the fruitfulness of state intervention in mortality decline – a concept that can be adopted in the developing countries where the socioeconomic factors slow down the pace of mortality changes.

The second aim of the analysis was to understand the changes in the mortality decline and improvement in life expectancy in different age and sex groups to figure out the possibility of mortality transition reaching an advanced stage in the state. Our speculation was that mortality reduction would be higher in the older age groups denoting the onset of advanced stages in the state. The analysis of TLE reveals that the age groups below 60 (except males at 40–60) have already reached their limit of mortality reduction in contributing to life expectancy. Thus, the possibility of a further mortality decline that can contribute to an improvement in life expectancy is confined to the older age groups. However, the analysis of Index of ARC in TLE found that the pace of TLE in the older age groups (40–60 and 60–80) is relatively lower than in youngsters, implying low mortality decline in the older age groups.

Median age at death and survival proportional to age 60 and 80 reflect the relative importance of reduction in old age mortality for further increasing life expectancy in the state. However, the contribution of different age groups to life expectancy until 1991–2000 shows that reduction in mortality in adults and the older age groups contributed <50 percent to increments in life expectancy. It indicates that the state has not yet entered into the advanced stage of mortality transition. Nevertheless, there is a recent overall tendency of increasing contribution from older age groups toward life expectancy. This could point to an emergence of this advanced stage, particularly among females.

The slow reduction in the mortality rates among adults and elders are associated with the ongoing epidemiological transition and mismatches in health policies in Kerala. The third aim of our analysis, the decomposition analysis on changes in life expectancy by the major causes of death is corroborating with this finding. Notably, the state is experiencing more deaths due to non-communicable diseases such as cardiovascular diseases, neoplasm, accidents, and injuries by shifting the dominance of deaths from infectious diseases and maternal and child deaths in the recent decades as part of ongoing epidemiological transition. These causes affected the adults and elders to a large extent. The shift in the causes of death necessitates a different level of healthcare mechanism. In other words, the post-1971 epidemiological stage in Kerala requires promotive and curative intervention in addition to maintaining the existing primary healthcare facilities. In view of this, any delay in the onset of the advanced stage of mortality transition is caused by lower mortality decline of adults and elders and raises concerns over the efficiency of the existing healthcare system in tackling the additional challenges.

At this juncture, the experience of developed countries such as the United States and western European countries can shed some light. The basic feature of advanced mortality reduction in these countries was the reduction in morbidity mainly as a result of a combination of preventive and health-promotive measures. Moreover, supplementing their successful primary healthcare model with a supportive intervention by providing accessible healthcare facilities and financial support through health insurance also contributed to their success. However, such focused strategies are not yet widely implemented in Kerala. Since Kerala already has a good network of primary healthcare services, the focus should shift to the health problems of adults and address concerns related to their morbidity and mortality. The current trends in disease pattern suggest that different strategies other than the typical primary healthcare intervention followed are necessary for addressing the emerging changes in the disease pattern in the state.

**Main findings**

- Health issues of infants, children, and mothers of reproductive age have been effectively addressed by various policies inKerala, leading to dramatic decreases in overall mortality and increase in life expectancy during the last century.
- Further mortality reduction is possible in adult and early old age groups, which could further increase life expectancy in Kerala.
- The limited reduction in mortality in the adult and early old age groups inKerala during the last century is mainly due to the effects of highly prevalent non-communicable diseases. This also indicates a slow progress towards the advanced stages of epidemiological transition.

**Key messages for action**

- Health status of adult and elderly men is unsatisfactory, particularly given the large differences in mortality observed between men and women in these age groups; future government policy should prioritise their health needs.
- High mortality and morbidity in Kerala are now mainly due to non-communicable diseases; planning and policy in healthcare should reflect these changes in disease patterns.
- Priority should be given to strategies for disease prevention and health promotion, in addition to existing curative healthcare services, in order to curb the challenges from non-communicable diseases.
